# Disease detection, epidemiology and outbreak response: the digital future of public health practice

**DOI:** 10.1186/s40504-018-0071-4

**Published:** 2018-04-01

**Authors:** Edward Velasco

**Affiliations:** Robert Koch Institute, Healthcare-associated Infections, Surveillance of Antibiotic Resistance and Consumption, Seestrasse 10, 13353 Berlin, Germany

## Abstract

Inequalities persist when it comes to the attention, resource allocation and political prioritization, and provision of appropriate, adequate, and timely health interventions to populations in need. Set against a complex socio-political backdrop, the pressure on public health science is significant: institutions and scientists are accountable for helping to find the origins of disease, and to prevent and respond effectively more rapidly than ever. In the field of infectious disease epidemiology, new digital methods are contributing to a new ‘digital epidemiology’ and are seen as a promising way to increase effectivity and speed of response to infectious disease and public health events. New types of health data and access to personal information that are available through diverse channels will continue to have wide implications for epidemiology and public health practice. The purpose of this short paper is to introduce the emerging backdrop of practical and ethical challenges for those involved within the practice of public health as they face increasing collaborations with those from fields that have not traditionally applied their methods to epidemiology.

## Despite public health advances, infectious diseases persist

One third of worldwide deaths are attributable to infectious diseases, which are the second cause of mortality and disability worldwide (Global Health Observatory (GHO) 2017).While many known infectious diseases are increasingly the focus of research institutions, private grant-making foundations and global health and development agencies, it is socio-political events and emergencies of public health importance that often drive priorities. Global investment in the areas of HIV/AIDS, TB and malaria have had widespread consequences with positive impact in many endemic and persistent areas of the world (WHO 2015). Despite this, there are emerging and still-unknown infectious causes—responses to a continuous physio-biological and environmental evolution that, despite advances, enable infectious diseases to persist both within and outside healthcare associated environments (Global Health Observatory (GHO) 2017). Additionally, inequalities persist when it comes to the attention, resource allocation and political prioritization, and provision of appropriate, adequate, and timely health interventions to populations in need.

## Science or technology: what leads in public health practice?

Set against a complex socio-political backdrop, the pressure on public health science is significant: institutions and scientists are accountable for helping to find the origins of disease, to prevent, and to respond effectively and more rapidly than ever. In the field of infectious disease epidemiology, new digital methods are contributing to a new ‘digital epidemiology’ and are seen as a promising way to increase effectivity and speed of response to infectious disease and public health events. Digital epidemiology is the use of digital means for the purposes of – and the monitoring, research, analysis, and decision making implicit in – disease surveillance.

Digital epidemiology has the same objective as traditional epidemiology, to enable practitioners to measure and describe infectiousness, based on a basic reproductive rate (R0) greater than 1 (accounting for number of cases (C), personal attributes (P), infectiousness of the particular disease (I), and time of exposure (T):

$$ \mathrm{R}0>1\ \mathrm{x}\ \left(\mathrm{C},\mathrm{P},\mathrm{I},\mathrm{T}\right) $$

Digital epidemiology superimposes another layer onto this, and also comprises information sources from technology, user-based data from the Internet, like social media, and also technologies linked to mobile devices (e.g. smartphone apps) and networks. Coupling traditional and digital sources of information can arguably allow for innovation in interpreting infectiousness, and faster and better infectious disease assessment and response from information on personal health metrics to aggregated information on people-to-people chains, such as those in larger outbreaks of global health importance. Biostatistics - the backbone of epidemiology - is accordingly shifting to encompass not only the traditional, strictly mathematical techniques like standardization, distribution and probabilities found within controlled datasets, but also increasingly includes methods from network theory, where relational complexity and algorithmic prediction is performed on less controlled, often electronic datasets.

## Public health surveillance: then and now

Individual data that is aggregated into health statistics and collected routinely is a major part of indicator-based surveillance: the standard approach to epidemiological surveillance as practiced traditionally by health scientists at local, regional, and national health agencies and public health departments. The methods commonly employed to conduct indicator-based surveillance have been widely validated and provide an established way to conduct official, continuous surveillance using reliable data for epidemiology (Velasco 2014). Because information often flows through official channels, personal data is often protected by agencies who usually comply with verified channels and are accountable to ensure privacy for individuals within a population. The classic example is data collected about an infection by a physician seeing a patient, who then reports a specific case to his local public health agency - depending on the (sometimes legally outlined) reporting requirements of the infectious diseases at hand. The information is passed on to state and national or international health monitoring agencies for eventual aggregation as part of routine collection of infectious disease statistics. Meeting high levels of verification and protection of data that flows through official channels means that aggregation is necessary for many diseases, and such data sets often lose context after aggregation. Additionally, information is slow to reach analytical stages, since it is reliant on previously developed detection filters, and often leaves traditional methods poorly equipped to detect unexpected emerging diseases or increasingly faster-spreading outbreaks.

In contrast to indicator-based surveillance, new surveillance methods that comprise digital epidemiology employ methods of analyzing individual-based information in addition to aggregated information. This information is often personalized, it is comprised of individual data and is rich in context and quickly available, since it is often derived from the person directly through their smartphone, apps and then digitally transferred for analytical processing via (often) unofficial avenues that are usually not directly related to public health authorities. Such data is available in larger amounts, and in the last years has been easier than ever to analyze. Xihong Lin, Chair of Biostatistics at Harvard, speaks of an “-omics revolution”: the drastic changes to computational science that have been enabled by cheaper and faster access to large datasets, whether from the bio-genome (data from our vast genetic and biological codes), the exposome (data on substances and experiences that we are exposed to) or the phenome (every possible disease outcome, including what is in a person’s medical record). “Traditional epidemiology and environmental studies are hypothesis-driven. Now we can generate new hypotheses directly from the data, matching pieces to access this huge puzzle to identify multiple causes of disease, treatment targets and prevention strategies…and it requires an open mind, curiosity, and creativity” (Lin 2015).

A personal message on popular social media applications may be filtered for key words, geolocation, or images; video content could possibly be linked to online profiles with access to personal biological and health data, such as data that is collected from performance monitoring watches or other sportswear. Of recent interest are data from DNA and *omics analytical websites, activity information (i.e. related to exercise and individual biostatistics like heart rate, blood pressure, and dietary patterns), commercial lab testing results that are outside traditional hospital and health insurance networks, and even emotional logs, like diaries and records on stress symptoms via apps. Vast amounts of data generated from healthcare and medicine are stored and can be used to explore risk factors and outcomes that could be extrapolated to health conditions. Possibilities for analysis of such data are also, as also mentioned by Dr. Lin, easier due to cheaper opportunities to collect very large amounts of data and the ability store them (think: cheaper computing for genomic sequencing) (Wylie and Davies 2015; Raj 2014).

Health and infectious disease related information increasingly passes from person-to-person through networks that enable information to flow directly to scientists without the assistance of physicians or other health officials, and accordingly their role has diminished. But this also means that specific data protections and privacy mechanisms may also be diminishing as innovative methods in contemporary epidemiology newly arise from the availability of more data from diverse sources other than healthcare professionals and public health authorities. There is a growing popularity of complex public health issues among new scientists from fields that have not traditionally applied their methods to epidemiology. One sees this in the emerging epidemiological work of physicists and network scientists. Their interest in using medical and health data, sometimes derived from consumer behavior (as found in research efforts by corporations using direct-to-consumer health products like health monitoring sportswear or DNA tests) has been a boon to exploring new challenges in an increasingly connected global chain of health events. Their research aims to show complex interactions of biological contagion and infectious sequelae among individuals who are interconnected in networks of various degrees of separation across the globe. Global mobility and network driven contagion phenomena aim to show where and when diseases emerge and are used to illustrate complex theories on pathogen origin and spread.

## Integrated data science is the future of public health

New types of health data and access to disease information that are available through diverse channels will continue to have wide implications for epidemiology and public health practice. New data will lead to new methods and tools, and transdisciplinarity in the use of those methods and tools will allow for new interpretation of the complex dynamics of infectious disease detection, epidemiology and outbreak mitigation. But this is not without practical, and ethical challenges for those involved with the practice of public health.

Facing challenges that would likely prohibit a vision for full privacy, public health practice should instead aim to include long term plans to address these challenges, which could enable self-determination and pooling of knowledge on scientific methods to handle information and data sensitivity on a rolling basis. This could help to improve the algorithms that process such data, but could also enable the field to remain dynamic in the face of ever faster and quickly evolving technologies. Scientists from all fields can collaborate on innovative approaches from collective, diverse participation, but they will also need to face new parameters of professional accountability.

There is widespread interest in legitimizing big data for the field of digital epidemiology. The concept of a “public health footprint” has been used to explore accountability when it comes to the direct and indirect effects of using healthcare data in the private sector, and it is just one example that might be applied to any institutions using such data (Podcast 2015). Bioethicists are working to keep up with what implications this public health footprint might have - especially in Europe - on privacy and protection. The science of digital epidemiology itself must be challenged, tested and validated. It remains unclear (and often proprietary) how corporations involved in digital epidemiology are aggregating data. Often, the challenge of using massive amounts of meta data is that it quickly loses context - a group or crowd effect can be described or effects can be overestimated, instead of describing an actual health reality, in much the same way data from traditional indicator-based surveillance can be compromised by too much time lost. The era of internet based data will need to evolve with mechanisms that bring back validation measures, and transparency that can continue to drive science. “Google collects data on human interaction and feeds back statistics on it, it’s a very rudimentary form of artificial intelligence,” opined Sandy Pentland of MIT at a recent talk in Berlin, and indeed there is a role for data curation in the digital sciences, and experts from all fields are able to participate in creating a structure for validation that ensures channels and filtration of data so that it remains relevant to the core epidemiological science of the basic reproductive rate. Finding a way to create such a validated structure is the challenge in contemporary public health practice.

Are regulatory contexts robust enough in their procedural-level mechanisms to ensure a responsible public health footprint, and to promote the adoption of advanced scientific methods for increasingly large, complex data environments? What governance structures beside regulation can be further developed? When it comes to digital data and equality, the question becomes ‘who is participating?’ Which segments of the population are contributing to data sets? Whose privacy is at stake? Who owns digital data and how much protection is required? To date, such questions remain unanswered.

## Conclusion

The overarching driving factor in epidemiology will remain the drive to end inequalities that persist for creation of and access to health interventions to affected populations. Methods are rapidly evolving to extrapolate data at an aggregated level that also remain beneficial to epidemiology, and technology is increasingly central to this the emergent area called digital epidemiology. But recent cyberattacks around the world show a critical need to address the sensitivity of data, and the importance of data protection: from keeping it true to protecting it from the potentially harmful effects of data transfer among technologies, apps and the methods used to analyze data. Public health surveillance has traditionally played a central role to validate data and has been accountable for ensuring rigor that depicts a reality that can inform political action. Public health practitioners will continue to be held to these parameters even as the uptake of technology increases within the scope of their work. But the real challenge for public health practitioners will be to accept innovation and the changing paradigm for public health science, which make infectious disease detection and response more complex than ever: this includes complex data collection on a wide array of health events, analysis of data from globalizing social and contagion chains, and the time and expertise that is necessary to make sense of digital data. All the diverse parties involved in public health surveillance must be invited to participate in access, distribution and protection of that data to create transparency, and to build structures for legitimacy. It is time to divest digital responsibility also to scientists in all relevant fields so that they are invested in managing open and vulnerable health information in the digital age. We can and will hold their work to the standards of public health practice, that.

*“the right to the highest attainable standard of health should be the cornerstone of any consideration of health and human rights”* (Hunt and Backman 2008).
